# Nutraceutical Antioxidants as Novel Neuroprotective Agents

**DOI:** 10.3390/molecules15117792

**Published:** 2010-11-03

**Authors:** Natalie A. Kelsey, Heather M. Wilkins, Daniel A. Linseman

**Affiliations:** 1 Department of Biological Sciences and Eleanor Roosevelt Institute, University of Denver, Denver, Colorado 80208, USA; E-Mails: nkelsey@du.edu (N.A.K.); heather.wilkins@du.edu (H.M.W.); 2 Research Service, Veterans Affairs Medical Center, Denver, Colorado 80220, USA

**Keywords:** natural products, oxidative stress, neurodegeneration, neuronal apoptosis, reactive oxygen species

## Abstract

A variety of antioxidant compounds derived from natural products (nutraceuticals) have demonstrated neuroprotective activity in either *in vitro* or *in vivo* models of neuronal cell death or neurodegeneration, respectively. These natural antioxidants fall into several distinct groups based on their chemical structures: (1) flavonoid polyphenols like epigallocatechin 3-gallate (EGCG) from green tea and quercetin from apples; (2) non-flavonoid polyphenols such as curcumin from tumeric and resveratrol from grapes; (3) phenolic acids or phenolic diterpenes such as rosmarinic acid or carnosic acid, respectively, both from rosemary; and (4) organosulfur compounds including the isothiocyanate, L-sulforaphane, from broccoli and the thiosulfonate allicin, from garlic. All of these compounds are generally considered to be antioxidants. They may be classified this way either because they directly scavenge free radicals or they indirectly increase endogenous cellular antioxidant defenses, for example, via activation of the nuclear factor erythroid-derived 2-related factor 2 (Nrf2) transcription factor pathway. Alternative mechanisms of action have also been suggested for the neuroprotective effects of these compounds such as modulation of signal transduction cascades or effects on gene expression. Here, we review the literature pertaining to these various classes of nutraceutical antioxidants and discuss their potential therapeutic value in neurodegenerative diseases.

## 1. Introduction

There are a wide variety of neurodegenerative diseases with distinct symptoms and pathologies. For many of these diseases, the vast majority of cases are sporadic and therefore, the challenge is to discover the underlying causes of neurodegeneration in order to prevent or slow these disorders. Oxidative stress is recognized as a common factor in many neurodegenerative diseases and is a proposed mechanism for age-related degenerative processes as a whole [1,2]. Numerous studies have provided compelling evidence linking neuronal oxidative stress to Parkinson’s disease (PD) [3,4,5,6,7], Alzheimer’s disease (AD) [8,9,10], amyotrophic lateral sclerosis (ALS) [11,12], and multiple sclerosis (MS) [13,14], to highlight but a few. 

Oxidative stress occurs when reactive oxygen species (ROS) accumulate in the cell, either from excessive production or insufficient neutralization, causing damage to DNA, lipids, and proteins. Mitochondria are both a major source and target for ROS. Mitochondria are the powerhouses of the cell; they have the essential function of generating cellular energy in the form of ATP. Without ATP the cell will become energy deprived and eventually die. The most effective way for a cell to produce ATP is through oxidative phosphorylation within the mitochondria via the electron transport chain (ETC). The ETC is not entirely efficient so there is a basal level of electron leak under even the most optimum of conditions. The inadvertent leakage of electrons and their reaction with molecular oxygen are major contributors to the production of cellular ROS. Moreover, ROS produced within mitochondria subsequently target the various components of the ETC (in particular, complexes I and III), resulting in a vicious feed forward cycle of enhanced generation of ROS, more severe ATP depletion, and ultimately cell death [15,16]. Various genetic mutations and environmental exposures can undoubtedly sensitize neurons to mitochondrial ROS production either by increasing the exogenous production of free radicals or decreasing endogenous antioxidant defense systems. 

Based on the premise that oxidative stress underlies a number of neurodegenerative diseases, the identification of novel antioxidants as potential therapeutics is a prolific area of neuroscience research [17]. Amongst the most studied categories of antioxidants, dietary polyphenols and other natural antioxidants have rapidly gained attention as viable candidates for clinical testing in neurodegeneration and acute neuronal injury such as stroke [18,19,20,21]. In this review, we focus on a variety of natural compounds (nutraceuticals) and their abilities to act as antioxidants and cell protectants in neuronal systems. Given that oxidative stress is a principal cause of neurodegenerative disease, effective natural antioxidants could provide novel and safe therapeutic options for these devastating disorders. 

## 2. Intrinsic Antioxidant Properties of Nutraceuticals

**Figure 1:**
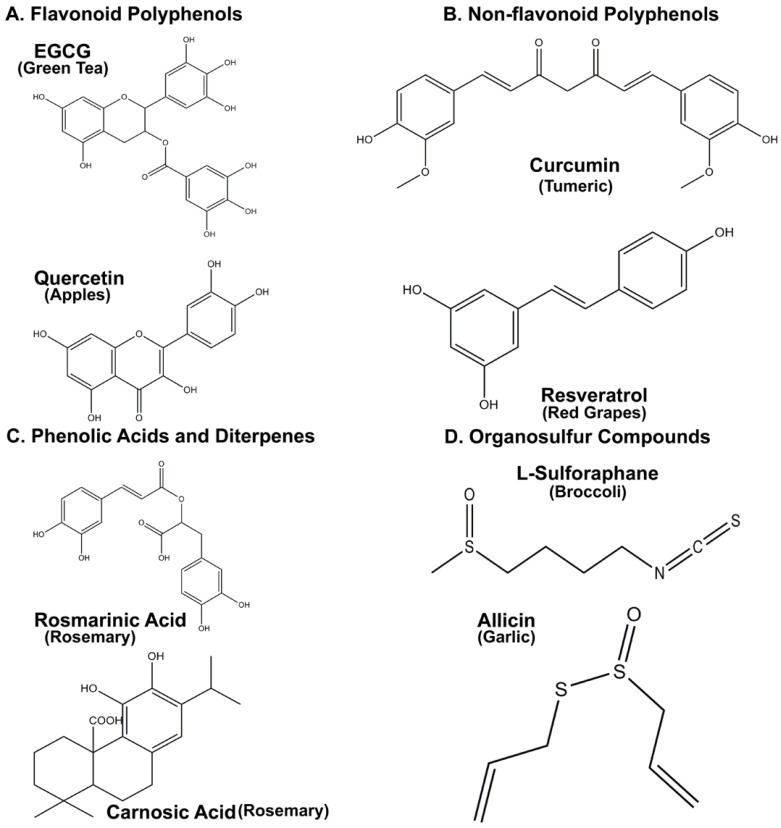
Chemical structures of various nutraceutical antioxidants.

There are many chemical classes of nutraceuticals found in all sorts of foods. Some nutraceuticals are well known, like epigallocatechin 3-gallate (EGCG) from green tea and resveratrol from grapes, while others are largely foreign to the lay consumer. The chemical structures of the natural compounds reviewed here are shown in Figure 1. Although these compounds differ structurally, each of them has been shown to have neuroprotective and antioxidant properties. 

A common method of determining intrinsic free radical scavenging activity is to use a cell free assay system with the radical 2,2-diphenyl-1-picryhydrazyl (DPPH). Resveratrol [22], carnosic acid [23], and rosmarinic acid [24] have each been shown to be effective scavengers of DPPH radicals. In contrast, allicin has been found to be a poor scavenger of peroxyl radicals while another garlic compound, 2-propenesulphenic acid, is a good scavenger of these radicals [25]. Additionally, EGCG has been shown to scavenge a wide variety of free radicals including superoxide, hydroxyl radical, hydrogen peroxide, and nitric oxide [26,27]. The intrinsic free radical scavenging activities of these nutraceutical antioxidants suggest that they may have potential utility in mitigating neuronal oxidative stress and neurodegeneration.

## 3. Neuroprotective Properties of Flavonoid Polyphenols

### 3.1. Epigallocatechin 3-Gallate (EGCG)

**Figure 2:**
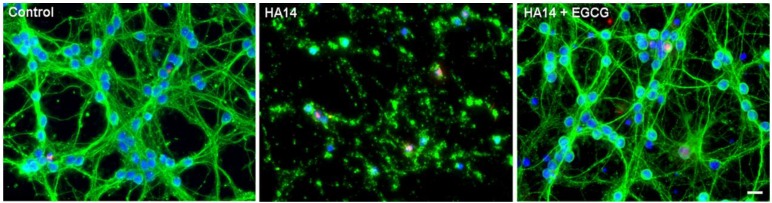
EGCG protects neurons from oxidative stress. Representative images of CGNs incubated for 24 hrs with the Bcl-2 inhibitor HA14-1 (15 M), HA14-1 + EGCG (25 M), or no treatment (Control). Immunocytochemistry was performed for β-tubulin (green) and active caspase-3 (red). Nuclei are stained with DAPI, blue. Scale bar; 10 microns.

EGCG (Figure 1A) is a flavonoid polyphenol and the main antioxidant compound found in green tea. EGCG displays neuroprotective effects in a variety of *in vitro* paradigms. Our own work has shown that EGCG selectively protects cultured rat cerebellar granule neurons (CGNs) from oxidative stress [28]. Figure 2 shows the dramatic effects EGCG has against oxidative stress in the CGN model. CGNs incubated with the Bcl-2 inhibitor, HA14-1 (ethyl 2-amino-6-bromo-4-(1-cyano-2-ethoxy-2-oxoethyl)-4*H*-chromene-3-carboxylate), undergo mitochondrial oxidative stress and intrinsic apoptosis [29,30]. Co-treatment with EGCG significantly preserves the microtubule network and prevents the apoptotic nuclear morphology of CGNs exposed to HA14-1 (Figure 2). 

Other studies have demonstrated similar results where EGCG significantly mitigates oxidative stress and neuronal death induced by hydrogen peroxide in motor neurons [31], N18D3 mouse neuroblastoma x dorsal root ganglion hybrid cells [32], spiral ganglion cells [33], and RGC-5 retinal ganglion cells [34]. EGCG similarly protects SH-SY5Y human neuroblastoma cells from amyloid precursor protein (APP), 3-hydroxykynurenine, or 6-hydroxydopamine (6-OHDA) toxicity [35,36,37], and rescues rat PC12 cells from serum withdrawal or paraquat-induced apoptosis [38,39]. In addition, EGCG reduces apoptosis caused by exposure of fetal rhombencephalic neurons to ethanol [40]. Furthermore, EGCG significantly reduces β-amyloid-induced toxicity in hippocampal neurons by inhibiting Aβ fibril formation and oligomerization [41,42]. Finally, EGCG rescues primary dopamine neurons from 1-methyl-4-phenylpyridinium (MPP+) toxicity [43]. Thus, EGCG exerts significant neuroprotective effects against a wide range of oxidative insults in a multitude of neuronal cell systems.

In addition to the neuroprotective effects of EGCG observed *in vitro*, this nutraceutical antioxidant also preserves neuronal survival and function in several *in vivo* models of neurodegeneration. For example, oral administration of EGCG protects mice from the dopaminergic toxicity caused by the Parkinson’s neurotoxin, 1-methyl-4-phenyl-1,2,3,6-tetrahydropyridine (MPTP). EGCG treatment prevents the MPTP-induced loss of dopamine neurons from the substantia nigra pars compacta and preserves striatal dopamine levels in mice [44]. In a similar manner, EGCG is protective in a mouse model of familial ALS. Oral dosing of EGCG to transgenic mice expressing a human G93A mutant SOD1 (Cu, Zn-superoxide dismutase) gene significantly delays symptom onset and moderately extends life span when compared to vehicle treated mice [45,46]. EGCG also reduces photoreceptor degeneration and improves motor function in a *Drosophila* model of Huntington’s disease [47]. Finally, oral administration of EGCG to Swedish mutant APP (APPsw) overexpressing transgenic mice substantially decreases amyloid plaque burden and reduces cognitive impairment [48]. Collectively, these findings indicate that EGCG may be a viable therapeutic candidate for chronic neurodegenerative diseases such as AD, PD, ALS, or Huntington’s [49,50]. Additionally, EGCG given by intraperitoneal injection to rats with induced spinal cord injury, reduces malondialdehyde (MDA) levels, TUNEL-positive staining, and lesion area, resulting in increased motor function [51]. This latter study suggests that EGCG may also be beneficial in episodes of acute neuronal damage such as spinal cord trauma. The principal mechanism of action of EGCG is probably antioxidant activity; however, the activation of specific protein kinase pathways (discussed below in section 7.2 and section 7.3) also appears to play a significant role in the neuroprotective action of this polyphenol.

### 3.2. Quercetin

Quercetin (Figure 1A) is a flavonoid polyphenol found in many common foods such as apples and capers. Like EGCG, quercetin has also been extensively studied in *in vitro* and *in vivo* neuronal models. *In vitro* studies in PC12 cells show that quercetin increases cell survival in the presence of hydrogen peroxide [52,53], linoleic acid hydroperoxide [54], and tert-butyl hydroperoxide [55]. Also, in C6 glioma cells quercetin alleviates oxidative stress induced by hydrogen peroxide or interleukin-1β [56,57]. In addition, in human SH-SY5Y neuroblastoma cells, quercetin protects against the PD toxin 6-OHDA. In another PD toxin model, MPP(+)-induced toxicity in mixed ventral mesencephalic cultures was significantly attenuated by quercetin treatment [58]. 

*In vivo* studies of quercetin effects on neurodegeneration have mostly focused on cognitive impairments, ischemia, and traumatic injury. Quercetin improves memory and hippocampal synaptic plasticity in models of impairment induced by chronic lead exposure [59]. In addition, quercetin is neuroprotective against colchicine administration, which similarly causes cognitive impairments [60]. In a rat ischemia model using middle cerebral artery occlusion, quercetin decreases the size of the ischemic lesion [61] and suppresses hippocampal neuronal death [62]. Finally, in a model of acute spinal cord injury, motor function was improved by administration of quercetin post-injury [63]. Cumulatively, these studies indicate that quercetin has the potential, like EGCG, to be developed into a novel therapy for neurodegeneration. 

## 4. Non-Flavonoid Polyphenols as Neuroprotective Agents

### 4.1. Resveratrol

Resveratrol (Figure 1B) is a polyphenolic antioxidant found in many kinds of grapes and is known mostly for its cardiovascular benefits [64,65]. However, resveratrol also demonstrates significant neuroprotective activity *in vitro* and *in vivo*. In various culture models, resveratrol protects organotypic hippocampal slices from oxygen-glucose deprivation [66], embryonic rat mesencephalic cultures from tert-butyl hydroperoxide [67], and CGNs from MPP(+)-induced toxicity [68]. *In vivo*, resveratrol significantly attenuates hippocampal neurodegeneration and learning impairment in the inducible p25 transgenic mouse model of AD and tauopathy [69]. Moreover, resveratrol also reduces oxidative damage and preserves striatal dopamine in the 6-OHDA rat model of PD [70]. The antioxidant activity of resveratrol plays a significant role in its neuroprotective mechanism of action as does its modulatory effects on sirtuins and protein kinases (discussed below in section 7.1 and section 7.3).

### 4.2. Curcumin

Research into the neuroprotective effects of the non-flavonoid polyphenol curcumin (Figure 1B), is less extensive than that for resveratrol. However, in Neuro2a mouse neuroblastoma cells infected with Japanese encephalitis virus, curcumin enhances cell viability by decreasing ROS and inhibiting pro-apoptotic signals [71]. *In vivo*, curcumin protects rats from focal cerebral ischemia induced by middle cerebral artery occlusion [72]. In addition, curcumin is neuroprotective against the MPTP-induced neurodegeneration of the nigrostriatal tract in mice and was shown to prevent glutathione depletion and lipid peroxidation induced by this toxin. Furthermore, curcumin displays an additive protective effect to that of catalase and SOD activities in the striatum and midbrain of MPTP-treated mice [73].

The studies noted above indicate that the non-flavonoid polyphenols, resveratrol and curcumin, each show beneficial effects in cell culture and *in vivo* models of neurotoxicity and neurodegeneration, respectively. Thus, these compounds may have promise as novel neuroprotective agents for clinical use.

## 5. Phenolic Acids and Diterpenes from Rosemary Demonstrate Significant Neuroprotective Properties

### Rosmarinic and Carnosic Acids

**Figure 3:**
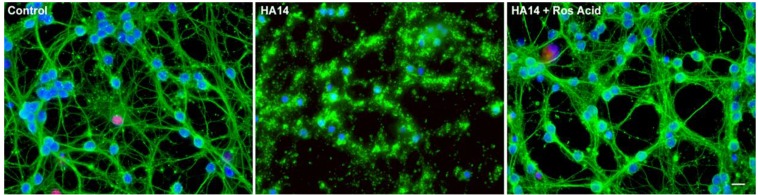
Rosmarinic acid protects neurons from oxidative stress. Representative images of CGNs incubated for 24 hrs with the Bcl-2 inhibitor HA14-1 (15 M), HA14-1 + rosmarinic acid (Ros Acid; 50 M), or no treatment (Control). Immunocytochemistry was performed for β-tubulin (green) and active caspase-3 (red). Nuclei are stained with DAPI, blue. Scale bar; 10 microns.

Phenolic acids and diterpenes constitute another family of nutraceutical antioxidants (Figure 1C). Several of these compounds are found in rosemary, with rosmarinic acid and carnosic acid being two of the most prominent antioxidants concentrated in this herb. Rosmarinic acid has been shown to scavenge the reactive nitrogen species, peroxynitrite, and various ROS [74,75]. As a free radical scavenger, rosmarinic acid is effective at protecting SH-SY5Y human neuroblastoma cells from hydrogen peroxide-induced oxidative stress and cell death [76]. In a similar experiment to the one shown above for EGCG (see Figure 2), we have demonstrated that rosmarinic acid provides dramatic neuroprotection in the CGN model against oxidative stress and mitochondrial apoptosis induced by the Bcl-2 inhibitor, HA14-1 (Figure 3). *In vivo* studies using mouse models of AD and ALS have shown that rosmarinic acid significantly alleviates memory impairment associated with Aβ neurotoxicity and significantly delays disease onset and prolongs lifespan in the G93A mutant SOD1 mouse model, respectively [77,78].

Carnosic acid, like rosmarinic acid, has been shown to be neuroprotective in both *in vitro* models of neuronal death and *in vivo* models of neurodegenerative disease. *In vitro*, carnosic acid activates the nuclear factor-erythroid 2-related factor 2 (Nrf2) transcription factor pathway (discussed in detail in the next section), and in this manner, protects neurons from oxidative stress [79]. *In vivo*, carnosic acid crosses the blood brain barrier and preserves reduced glutathione levels in the brain protecting it against injury induced by middle cerebral artery ischemia/reperfusion [79]. Collectively, these findings suggest that the phenolic acids and diterpenes concentrated in rosemary may provide a novel class of neuroprotective agents for future theraputic development.

## 6. Organosulfur Compounds as Inducers of Endogenous Antioxidant Defenses

### 6.1. Allicin and L-Sulforaphane

The last class of nutraceutical antioxidants to be discussed in this review includes the organosulfur compounds, allicin and L-sulforaphane (Figure 1D). Allicin is highly enriched in garlic, and garlic extract is used more often than pure allicin in many studies. One such study examined the effects of garlic extract on brain synaptosomes isolated from young *versus* old rats. In synaptosomes isolated from young rats, under both control and hydrogen peroxide-induced oxidative stress conditions garlic extract significantly decreased the production of 8-iso-prostaglandin F_2α_ (8-iso-PGF). 8-iso-PGF is a modified, unsaturated fatty acid released from the plasma membrane under oxidative stress. In contrast, aged rat brain synaptosomes only showed inhibition of 8-iso-PGF release at the highest dose of garlic extract studied and specifically under conditions of oxidative stress [80]. In a cell free *in vitro* study, garlic extract directly inhibited caspase-3, the executioner protease of the apoptotic cascade [81]. Thus, the neuroprotective mechanism of garlic appears to be two-fold; it depends on its capacity to suppress oxidative stress and its potential to inhibit caspase-3 and prevent apoptosis. The potential neuroprotective effects of garlic in the context of AD are reviewed elsewhere [82]. 

**Figure 4:**
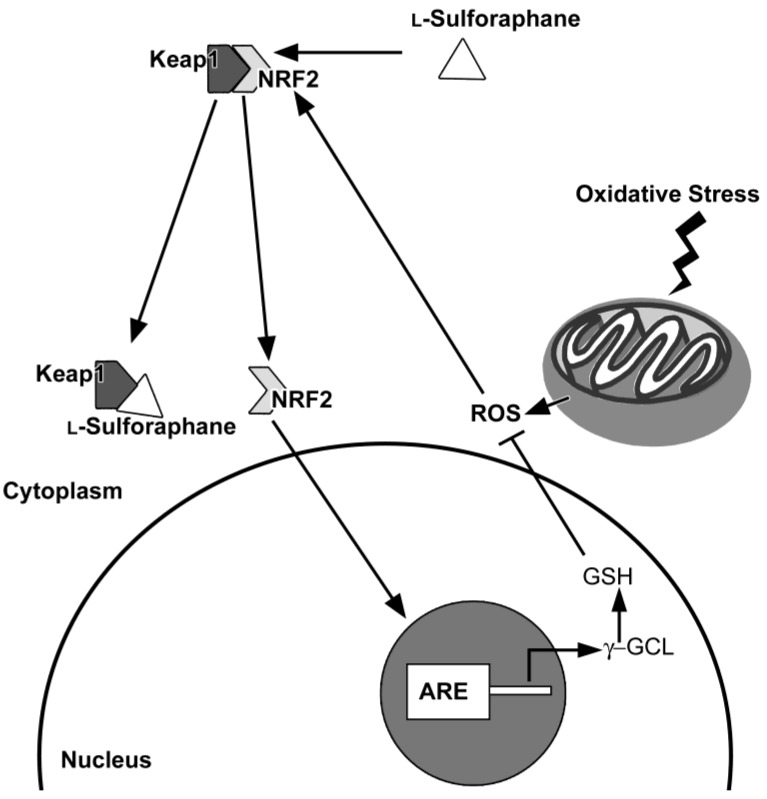
Activation of the Nrf2 transcription factor pathway by L-sulforaphane. The schematic shows a general mechanism by which ARE-mediated gene transcription is induced.

L-Sulforaphane is an isothiocyanate compound found in broccoli and other cruciferous vegetables which has also been used as a neuroprotectant. Dopaminergic neurons, which are affected in PD, produce toxic dopamine quinone and ROS when exposed to 6-OHDA [83]. Dopamine quinone-induced neuronal death is markedly inhibited by pretreatment with L-sulforaphane [84]. Additionally, neurons undergoing hydrogen peroxide-induced oxidative stress in a mixed neuron-astrocyte culture system are protected through stimulation of the Nrf2-antioxidant response element (ARE) transcriptional pathway, which L-sulforaphane has been shown to activate [85]. L-Sulforaphane activates this pathway by causing the dissociation of the negative regulator, kelch-like ECH associating protein 1 (Keap1), from Nrf2, as shown in Figure 4 (discussed in detail below). Finally, in a rat organotypic nigrostriatal tissue slice model, L-sulforaphane mitigated dopaminergic neuronal loss induced by 6-OHDA [86].

### 6.2. The Nrf2/ARE Antioxidant Pathway as a Target of Nutraceuticals

The above studies indicate that the sulfur-containing nutraceuticals, allicin and L-sulforaphane, demonstrate neuroprotective effects in a number of *in vitro* systems. Although these compounds may have some direct antioxidant effects that have yet to be elucidated, their principal mode of neuroprotection is indirect via activation of endogenous antioxidant systems, including gene targets of the Nrf2/ARE transcription factor pathway. 

ROS created during normal cellular respiration must be neutralized by cellular antioxidant defenses before these free radicals have the opportunity to damage the cell. As previously discussed, ROS become a major problem for the cell when there is an imbalance between ROS created and ROS neutralized. As the cell’s balance of ROS and antioxidants becomes disparate, oxidative stress occurs which can act as a trigger for apoptosis and other modes of cell death. The ETC within the mitochondria is a major source of ROS production within a cell. For this reason, it is important to have antioxidants like glutathione peroxidase and SOD located within the mitochondria. 

Glutathione peroxidase, SOD, and other endogenous antioxidants are critical for cell survival. In addition, transcription factors for these antioxidant genes, like Nrf2, are equally essential because they regulate the expression of these key antioxidants. In response to oxidative stress, Nrf2 induces a variety of antioxidant genes by recognizing an ARE binding site within their promoter regions [87]. Some key antioxidant genes induced by Nrf2 include γ-glutamylcysteine ligase (GCL), the rate limiting enzyme in the synthesis of glutathione (GSH), MnSOD (SOD2), and heme oxygenase, to name a few [88]. As a result, this pathway has been identified as a promising therapeutic target for neurodegenerative diseases [89]. Nrf2 is normally sequestered in the cytoplasm by Keap1, which must be dissociated in order for Nrf2 to translocate into the nucleus and promote gene transcription. The general mechanism of activating Nrf2 is demonstrated in Figure 4 and reviewed by Kobayashi and Yamamoto [90]. 

**Figure 5:**
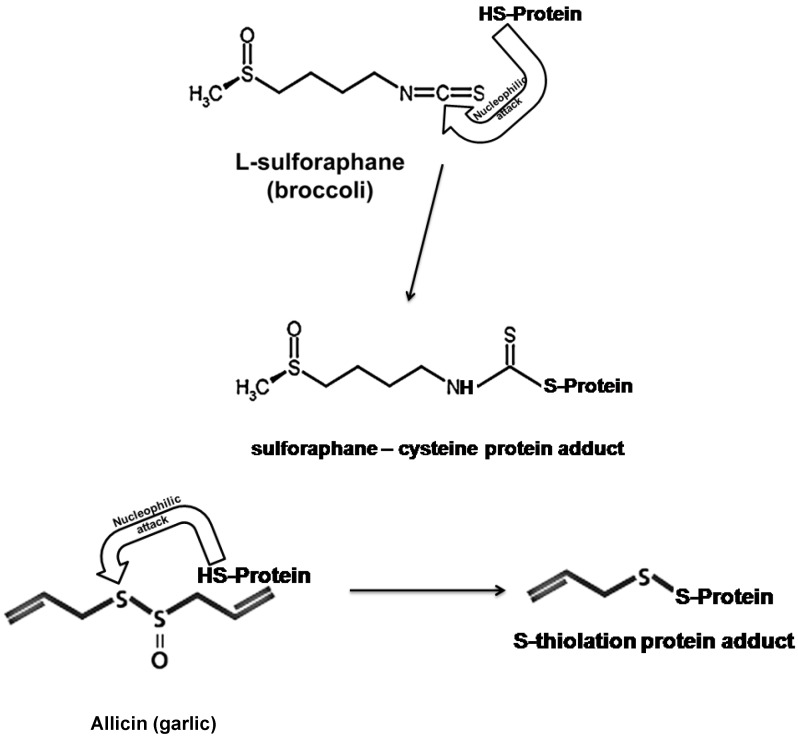
Proposed chemical mechanism by which organosulfur compounds dissociate Keap1 from Nrf2. HS-Protein = critical Cys residues on Keap1 which are essential for its ability to suppress Nrf2 activity. Modified from Hong *et al.* [91] and Rabinkov *et al.* [92].

The organosulfur compounds, allicin and L-sulforaphane, share the unique ability to activate Nrf2 [91,92,93]. This common attribute is derived from the fact that each of these compounds has an electrophilic center which can serve as an attack site for nucleophiles, such as specific protein sulfhydryl groups present on Keap1 (Figure 5). Indeed, the mechanism of Nrf2 activation by L-sulforaphane has been demonstrated to involve disruption of the Nrf2-Keap1 interaction due to modification of critical Keap1 cysteine residues [91,94,95]. The ability of these organsulfur compounds to induce Nrf2-ARE-dependent gene transcription suggests that this pathway is essential for their neuroprotective effects.

Nrf2 has been meticulously investigated in order to elucidate its role in antioxidant gene regulation. It has been shown to be neuroprotective in many different paradigms of neuronal injury or neurodegeneration. For example, an increase in Nrf2 activity protects SH-SY5Y human neuroblastoma cells from oxidative damage induced by the PD neurotoxin, 6-OHDA [96]. 6-OHDA was again used in both *in vivo* and *in vitro* models to demonstrate Nrf2 neuroprotection [86,97].

Mixed neuron/astrocyte cultures from mice are another model in which Nrf2 induction is protective against oxidative stress [85,98]. Nrf2 activation mitigates dopamine neuron loss and striatal dopamine depletion in the MPTP mouse model of PD [99]. In addition to Nrf2 activation being neuroprotective in the previously mentioned PD models, a transgenic AD mouse model showed attenuated Aβ toxicity following either adenoviral Nrf2 expression or induction of Nrf2 by tert-butylhydroquinone [100]. The critical importance of Nrf2 in controlling oxidative stress is further demonstrated by the enhanced oxidative stress and early embryonic lethality observed in combination Nrf1/Nrf2 knockout mice [101]. Nrf2 knockout alone is not embryonic lethal but does enhance the susceptibility of these animals to oxidative stress [89]. Finally, Johnson and colleagues have shown that Nrf2 induction specifically in *astrocytes* is sufficient to rescue neurons *in vivo* from death induced by mutant SOD1, MPTP, or malonate-induced complex II inhibition [102,103,104]. Given the striking neuroprotective effects of Nrf2 activation, it is reasonable to assume that nutraceutical Nrf2 inducers, like allicin and L-sulforaphane, may provide significant therapeutic benefit against neurodegeneration. 

## 7. Alternative Mechanisms of Neuroprotection Attributed to Nutraceuticals

### 7.1. Sirtuins

**Figure 6:**
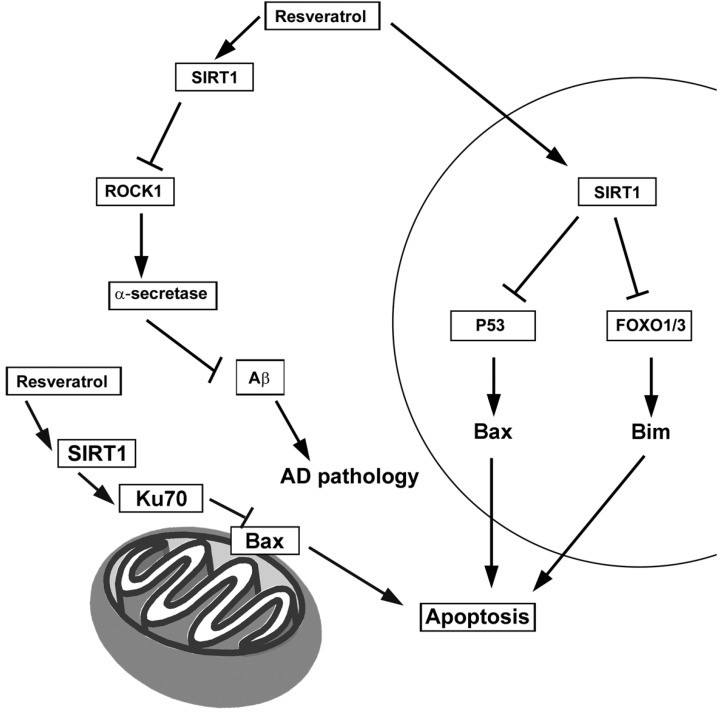
Pro-survival effects of SIRT1. The schematic demonstrates the downstream consequences of nutraceutical activation of SIRT1.

The sirtuin (SIRT) proteins are a part of the histone deacetylase family and they possess (NADH)-dependent deacetylase activity. SIRT1 is a homologue of the yeast gene, silent information regulator two (Sir2), which is linked to longevity. An extra copy of the Sir2 gene in yeast can mimic a calorie-restricted diet, extending lifespan [105]. In a similar manner, caloric restriction delays neurodegenerative disease onset. Qin *et al*. showed that caloric restriction activated SIRT1 in the brains of AD model Tg2576 mice, and reduced amyloid neuropathology [106]. Furthermore, they showed that expression of SIRT1 in either primary Tg2576 neuronal cultures or CHO cells expressing APPsw significantly attenuated Aβ peptide formation. SIRT1 has also been linked to alleviating Aβ toxicity in cortical neuron/glial co-cultures [107]. There is still much to learn about how nutraceuticals, like resveratrol, induce SIRT1. Regardless of whether the mechanism of SIRT1 activation by resveratrol is direct [108] or indirect [109,110], induction of SIRT1 appears to be a principal mechanism underlying the neuroprotective effects of this polyphenol [111,112,113]. The putative pro-survival effects of SIRT1 activation are multifaceted and involve the inhibition of Aβ peptide generation, suppression of Bax-dependent apoptosis, and repression of multiple pro-apoptotic transcription factors (Figure 6) [114,115,116,117,118].

### 7.2. Protein Kinase C (PKC)

**Figure 7:**
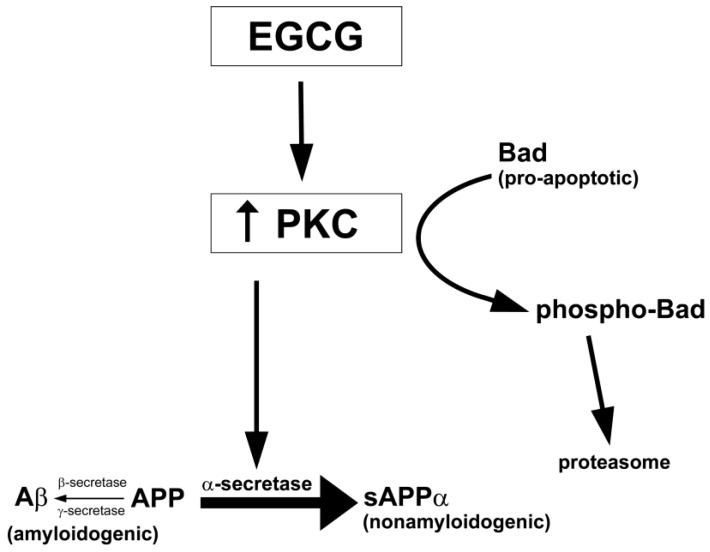
PKC is a key mediator of EGCG neuroprotection. The schematic shows the effects of PKC activation downstream of EGCG on the processing of APP being skewed towards the nonamyloidogenic product, sAPP, and the phosphorylation and targeting of pro-apoptotic Bad for degradation.

PKC is another protein involved in a myriad of signaling pathways including cell survival and programmed cell death [119]. In rat hippocampal neurons it was shown that resveratrol activates a PKC pathway which protects these neurons from Aβ toxicity [120]. EGCG is also known to activate a pro-survival PKC pathway. EGCG activation of PKC, via enhanced phosphorylation of this kinase, underlies its neuroprotective effects in SH-SY5Y and PC12 cells against Aβ toxicity [121]. The beta/gamma secretase-dependent processing of APP to the toxic Aβ peptide forms the basis of the pathophysiology underlying AD. There are however nontoxic processing pathways for APP, one of which is the alpha secretase-dependent production of nonamyloidogenic sAPPα. EGCG augments this nontoxic processing pathway through PKC activation [121]. PKC activation has also been implicated in EGCG neuroprotection from serum withdrawal in PC12 cells [122] and 6-OHDA toxicity in SH-SY5Y cells [35]. Finally, Kalfon *et al*. have connected EGCG to the PKC-mediated degradation of pro-apoptotic Bad in SH-SY5Y neuroblastoma cells [123]. Thus, the activation of PKC by EGCG may play as significant a role in its neuroprotective mechanism of action as its intrinsic antioxidant capacity (Figure 7). There are many isozymes of PKC that have been investigated individually for their neuroprotective effects. Specifically, PKC_epsilon_ overexpression has been shown to reduce amyloid plaque burden and Aβ levels in human APP transgenic mice [124].

### 7.3. Other Protein Kinases

**Figure 8:**
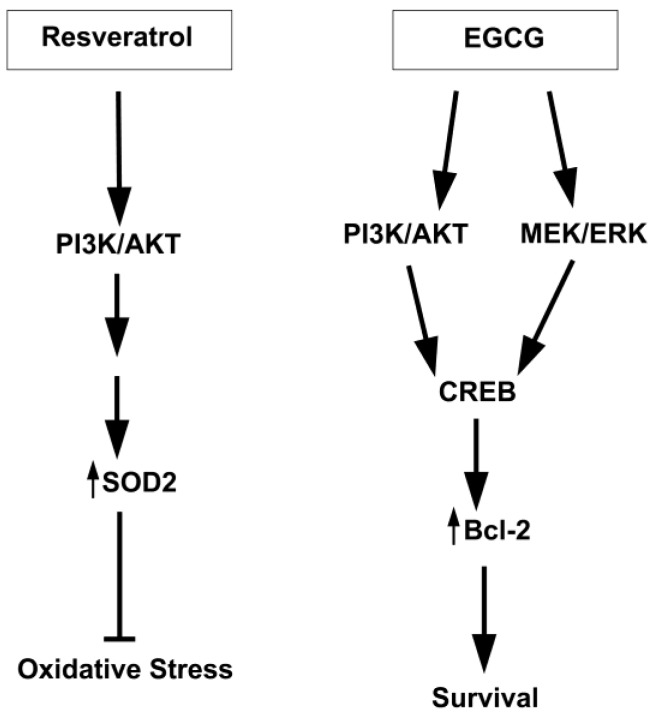
Modulation of pro-survival protein kinase pathways by nutraceuticals.

There are a number of additional signaling cascades that have been shown to be modulated by nutraceutical antioxidants including the predominantly pro-survival MEK/ERK and PI3K/AKT pathways, reviewed by Spencer [125]. For instance, resveratrol protects HT22 hippocampal cells from glutamate-induced oxidative stress via a PI3K/AKT-dependent induction of SOD2 [126]. Similarly, EGCG rescues retinal ganglion cells from axotomy-induced injury through activation of both PI3K/AKT and MEK/ERK pro-survival pathways [127]. Downstream of each of these pathways lies the transcription factor, cAMP-response element binding protein (CREB), which can induce the expression of key pro-survival genes like Bcl-2 [128,129]. Consistent with a role for this pathway in the neuroprotective effects of nutraceuticals, long term administration of green tea catechins in drinking water significantly increased CREB activity and decreased Aβ oligomer production in a mouse model of early onset deficits in learning and memory [130]. The characteristic of nutraceuticals to modulate key pro-survival kinase pathways likely plays a significant role in their neuroprotective actions (Figure 8).

## 8. Conclusions

Nutraceutical antioxidants have strong scientific support to be developed as novel therapies for neurodegenerative diseases. Many of these natural antioxidants are not only active scavengers of free radicals but also act as modulators of pro-survival or pro-apoptotic signaling pathways. As a result, these compounds may have a greater potential for therapeutic success than drugs with only one mechanism of action. The multiple modes of action of nutraceuticals to mitigate oxidative stress and promote neuronal survival signals likely underlie their effectiveness in so many *in vitro* and *in vivo* models of neuronal injury and neurodegenerative disease. Although individual neurodegenerative diseases manifest in distinct neuronal cell types, oxidative stress and suppression of neuronal survival signals are common to many of these pathological conditions and appear to be highly relevant targets for treatment. 

Overall, neurodegenerative diseases lack effective treatment options for patients. AD and PD receive the most attention through extensive funding and research, yet even these diseases have only palliative therapies available and none that significantly slow or halt the underlying pathology of the disease. Others, like ALS, have an even worse prognosis with death occurring typically 2-5 years after diagnosis and only one FDA approved drug, Riluzole, which is minimally effective and only prolongs life by two-to-three months. Nutraceutical antioxidants may be the best options for these patients in the short term since they are subject to fewer regulations than traditional pharmaceuticals and therefore, could be made available to patients much more rapidly than new prescription drugs. 

Finally, a testament to the tremendous potential of nutraceutical antioxidants as novel therapeutics for neurodegeneration includes the recent initiation of several clinical trials with these compounds. EGCG is currently being tested in Phase II trials for PD (Xuanwu Hospital, Beijing, China) and early stage AD (Charite University, Berlin, Germany). Similarly, resveratrol is being tested in a Phase II trial to improve memory performance in the elderly (McKnight Brain Institute, University of Florida) [131]. Lastly, the safety and tolerability of curcumin is being investigated in patients with AD in a Phase II study [132]. The future appears to hold much promise for nutraceutical antioxidants to provide significant therapeutic benefits to patients suffering from neurodegenerative diseases. Research, medical, and patient communities eagerly await the results of these initial clinical trials with this novel class of neuroprotective compounds.
